# Comparison of biotechnological culture of hypoxia-conditioned rat mesenchymal stem cells with conventional *in vitro*culture of normoxia-conditioned rat mesenchymal stem cells for testicular failure therapy with low libido in rats

**DOI:** 10.14202/vetworld.2019.916-924

**Published:** 2019-06-28

**Authors:** Erma Safitri, Mas’ud Hariadi

**Affiliations:** 1 Department of Veterinary Reproduction, Faculty of Veterinary Medicine, Universitas Airlangga, Surabaya 60115, Indonesia; 2 Stem Cells Research Division, Institute Tropical Disease, Universitas Airlangga, Surabaya 60115, Indonesia

**Keywords:** hypoxia-conditioned rat mesenchymal stem cells, low libido, normoxia-conditioned rat mesenchymal stem cells, rat, testicular failure

## Abstract

**Aim:**

Biotechnological culture of hypoxia-conditioned (CH) rat mesenchymal stem cells (rMSC-CH) for testicular failure therapy with low libido improves the functional outcome of the testicle for producing spermatogenic cells and repairs Leydig cells in rats (*Rattus norvegicus*).

**Materials and Methods:**

In the first group (T1), rats with testicular failure and low libido were injected with normoxia-conditioned (CN) rMSCs (21% oxygen); in the second group (T2), rats with testicular failure and low libido were injected with rMSC-CH (1% oxygen); in the negative control group (T−), rats with normal testis were injected with 0.1 mL phosphate-buffered saline (PBS); and in the sham group (TS), rats with testicular failure and low libido were injected with 0.1 mL of PBS.

**Results:**

Vascular endothelial growth factor expression, as the homing signal, in the groups T2, T−, T1, and TS was 2.00±0.5%, 2.95±0.4%, 0.33±0.48%, and 0±0%, respectively. The number of cluster of differentiation (CD)34^+^ and CD45^+^ cells in the groups T− and TS was <20%, whereas that in T1 and T2 groups was >30% and >80%, respectively, showing the mobilization of hematopoietic stem cells (HSCs). The number of spermatogenic cells (spermatogonia, primary spermatocytes, secondary spermatocytes, and spermatid) decreased significantly (p<0.05) in TS compared with that in T−, T1, and T2, whereas that in T2 did not show a significant (p>0.05) decrease compared to that in T−. The improvement in libido, based on the number of Leydig cells producing the hormone testosterone for libido expression, did not increase in T1, whereas T2 was able to maintain the number of Leydig cells significantly compared to that between TS and T1.

**Conclusion:**

rMSC-CH culture for testicular failure with low libido showed improvement in the functional outcome of the testicle and in repairing Leydig cells.

## Introduction

Oligospermia, a failure of the testicle, represents a major reproductive health problem in males, causing low fertility and libido [1,2]. Oligospermia is a condition in which low numbers of spermatozoa are produced by testicular seminiferous tubules, resulting in infertility in males, meaning failure to reproduce [3]. Oligospermia for male is the main cause of infertility with varying etiologies, such as heredity, trauma, neoplastic tumor, and degenerative disorders due to malnutrition [4].

Transplantation therapy with mesenchymal stem cells (MSCs) derived from the bone marrow has yielded very promising results in regeneration of spermatogenesis process in testicular tissue with infertility issues, such as oligospermia [5]. The efficacy of the therapy was limited due to low viability of stem cells after transplantation [6,7], which is due to the conventional *in vitro* culture of normoxia-conditioned rat MSCs (rMSC-CN) with oxygen concentration of >21%. The conventional culture causes cell senescence [8], apoptosis [9], and gene mutation, such as G: C to T: A [10]. Some researchers showed that >93% of stem cells perish between 1 and 7 days after transplantation [11-15]. Therefore, stem cells are required in high quantities with several booster doses for the effectiveness of therapy using conventional culture like normoxia-conditioned (CN), thus considerably increasing the cost [16]. To remain viable, *in vitro* culture of stem cells must be adapted for a niche or microenvironment wherein the stem cells reside in the bone marrow. In normal conditions, the niche of stem cells in the bone marrow is in a low oxygen concentration conditioned hypoxia (CH)] [5,8,10]. Therefore, biotechnological modification of rMSC-CH *in vitro* culture is required for homing signal and mobilization of stem cells to improve testicular function for producing sperms. The homing signal of stem cells in the testicle tissue is based on the expression of vascular endothelial growth factor (VEGF), whereas mobilization is based on the expression of cluster of differentiation (CD) such as CD34^+^, CD45^+^, and CD105^−^ cells [5-7].

The objective of the study was utilization of biotechnological culture of rMSC-CH for testicular failure therapy with low libido. It was revealed that biotechnological culture of rMSC-CH improved the functional outcome of the testicle for producing spermatogenic cells and repairing Leydig cells of rat (*R. norvegicus*).

## Materials and Methods

### Ethical approval

Animal studies were performed using a protocol approved by the Animal Care and Ethical Clearance Committee of Faculty of Veterinary Medicine, Universitas Airlangga, and were in conformance with the guidelines of the National Research Council (239-KE) through ethical seminar. The research was conducted at the laboratory in the Institute of Tropical Diseases and Faculty of Veterinary Medicine, Universitas Airlangga.

### Isolation and culture of stem cells

MSCs were collected from the bone marrow of iliac crest of male Wistar rats (*R. norvegicus*) at 3 months of age. The aspirate was collected in heparinized tubes and stored at 4°C for transportation to the laboratory at the Institute of Tropical Diseases, Universitas Airlangga, for *in vitro* culturing [17].

The aspirate of MSCs was collected in 15-mL heparin tube (Z181099, Sigma Aldrich^®^, Burlington, Massachusetts, USA), which were previously filled with 3 mL of α-modified Eagle medium (α-MEM) (M0894, Sigma Aldrich^®^, Burlington, Massachusetts, USA). The aspirate was transferred to a 15-mL sterile blue cap tube and sterile 1× phosphate-buffered saline (PBS; MFCD00131855, Sigma Aldrich^®^, Burlington, Massachusetts, USA) was added to a total volume of 10 mL. The tube with the aspirate solution was then rinsed twice with 5 mL of PBS. The diluted sample was added with equal volume of Ficoll (F9378, Sigma Aldrich^®^, Burlington, Massachusetts, USA) at room temperature of 37°C in a separate 15-mL tube. Furthermore, each aspirate was mixed with Ficoll before centrifugation (Sorvall™ MX Series Floor Model Micro-Ultracentrifuge, Thermo Fisher, Grand Island, USA) at 1600 rpm [287 relative centrifugal force (rcf)] for 15 min at room temperature of 37°C. After centrifugation, mononucleated cells were collected in the form of “buffy coat” located on the surface of Ficoll–PBS using a sterile Pasteur pipette and taken in a 15-mL tube (Sigma Aldrich^®^, Burlington, Massachusetts, USA).

The sample was diluted with PBS to a total volume of 15 mL, with the tube being turned 3–5 times as a means of achieving an even mix. At the next stage, centrifugation at 1600 rpm for 15 min at room temperature of 37°C was performed for 10 min at a speed of 1600 rpm (287 rcf). Before heating, the supernatant was discarded, and the cells were resuspended in 6 mL of α-MEM (M0894, Sigma Aldrich^®^, Burlington, Massachusetts USA). The cell suspension was placed in 10-cm^2^ plate (Falcon™, Thermo Fisher Scientific, Pittsburgh, PA, USA) and incubated at 37°C for 24 h in a humidified atmosphere containing 5% CO_2_ until cells adhered on the surface of the plate. After 24 h, media and non-adherent cells were discarded. The adhered cells were rinsed twice using 5 mL of PBS and shaken before heating the culture. The supernatant was discarded, and the plate was washed again twice with PBS. After 10 min, 10 mL of fresh α-MEM (M0894, Sigma Aldrich^®^, Burlington, Massachusetts, USA) was added to the dish before incubation. The cells were incubated at 37°C with 5% CO_2_, and the culture was observed daily using an inverted microscope. (MXD-400 Phase Contrast, Nanjing BW Optics and Instrument Co., Ltd)

Every four days, the media were discarded, and cells were rinsed with 5 or 10 mL of 1× PBS before heating. PBS was subsequently discarded, and the dish was filled with 10 mL of fresh α-MEM (M0894, Sigma Aldrich^®^, Burlington, Massachusetts, USA). The cells were cultured continuously until approximately 75%–80% confluence was attained. The cells were then passaged into several dishes for subculture [17].

Passaging was conducted three times, and then cells were divided into two groups, viz., rMSC-CH treatment with 1% O_2_ in a hypoxia chamber inside a 5% CO_2_ incubator and rMSC-CN with 21% O_2_; both were incubated for four days. The three gases needed for 1% low oxygen precondition are 5% CO_2_ to flow at 37°C in the incubator, 100% O_2_ only for calibration, and N_2_. N_2_ was used to replace O_2_ to maintain the desired concentration.

### Animal model of testicular failure and low libido

Testicular failure in rats was obtained by fasting for five days, but drinking water was provided *ad libitum*. [1]. The condition of fasting for five days induced testicular failure in the rats because the function of adrenal cortex was suboptimal in producing dehydroepiandrosterone (DHEA) due to malnutrition. Low levels of DHEA in the blood can be a cause of fatigue and decreased sperm concentration. DHEA is the most potent precursor of steroid hormones, such as testosterone, which is produced by the renal adrenal cortex [18] and Leydig cells of testis [3]. Low testosterone production can lead to decreased spermatogenesis and libido in males.

The animal model used in this study was male Wistar rats (*R. norvegicus*), 250–300 g body weight, 3 months of age, and healthy condition. Individual rats were placed in plastic cages at the Animal Laboratory Experimental, Faculty of Veterinary Medicine, Universitas Airlangga.

### Biotechnological culture of rMSC-CH compared with rMSC-CN culture

Biotechnology stem cell treatment was performed by injecting MSCs directly from either conditioned rMSC-CH or rMSC-CN in the medial part of both testes, following anesthesia using ketamine (87 mg/kg of body weight) and xylazine (13 mg/kg of body weight) at 10-15 min after simultaneous injection and lasting for 15-30 min, followed by a relatively long period of immobility (mean, 3.8 h) and reduced responsiveness to external stimuli [19].

MSCs injected into male rats induced testicular failure, such as oligospermia with low libido, compared with that in the negative and sham group. In the first group (T1), rats with testicular failure and low libido were injected with 200 million rMSC-CN/rat from high oxygen culture (21% O_2_); in the second group (T2), rats with testicular failure and low libido were injected with 200 million rMSC-CH/rat from hypoxia culture (1% O_2_); in the negative control group (T−), rats with normal testicle were injected with 0.1 mL of PBS; and in the sham group (TS), rats with testicular failure and low libido were injected with 0.1 mL of PBS.

After two cycles of spermatogenesis, male rats were operated to collect the testicle tissue. Immunohistochemistry (IHC) analysis was performed to determine the expression of VEGF as the homing signal of stem cells in the testicle tissue. Flow cytometry was performed to calculate the number of CD34^+^, CD45^+^, and CD105^−^ cells, as an indicator of stem cell mobilization, to improve testicular disorders.

The improvement in the testicle for producing sperm cells was observed by histopathological analysis by hematoxylin-eosin (HE) staining (B8438, Sigma Aldrich^®^, Burlington, Massachusetts, USA), and then the number of spermatogenic cells (spermatogonia, primary spermatocytes, secondary spermatocytes, and spermatid) and Leydig cells were calculated.

### IHC analysis for VEGF expression

IHC analysis was performed to determine VEGF expression. Histological preparation was done before IHC. A transverse incision was made in the paraffin block containing the testicular tissue. Further examination was performed by determining the external expression of VEGF by IHC using VEGF monoclonal antibody (A183C 13G8, Thermo Fisher Scientific, Pittsburgh, PA, USA) with PBS (MFCD00131855, Sigma Aldrich^®^, Burlington, Massachusetts, USA) dilution. VEGF expression was observed using a regular light microscope (Oxford BCM 400, monotaro.id, Indonesia) under 200× magnification, and the expression of each variable was indicated by the number of cells with the development of brownish discoloration of the chromogen in each incision [20]. The cells were observed using a regular luminescence microscope Nikon H600L (Nikon Instuments Inc., America) equipped with a digital camera (DS Fi2, 300 megapixels) and image processing software and cell count Nikon Image System.

### Flow cytometry

Flow cytometry (BD FACSCalibur™, BD Biosciences, San Jose, USA) observation of stem cell mobilization was based on the calculated number of CD34^+^, CD45^+^, and CD105^−^ cells. After the treatment, whole blood was collected in a heparin tube via cardiac puncture to prevent coagulation. Flow cytometric observation revealed the numbers of CD34^+^, CD45^+^, and CD105^−^ cells. In brief, whole blood was centrifuged at 6000 rpm (4032 rcf) at 4°C for 15 min. The cellular precipitate obtained after centrifugation was mixed with cytoperm/cytofix (554714, BD Cytofix/Cytoperm™, BD Biosciences, San Jose, USA) at twice the volume of that of the obtained cell volume. This mixture was then centrifuged at 1600 rpm for 15 min at room temperature of 37°C.

To separate the supernatant and pellet, BD wash was added to the pellet at four-times the volume of the obtained cell volume from the first centrifugation, and then lysis buffer was added at twice the volume of the first obtained cell volume. Subsequently, the labeled antibody was added to each sample, and five tubes were arranged and processed simultaneously: (1) single staining with CD34 PE (Rabbit Anti-CD34/HCAM/PGP1 Polyclonal Antibody, PE-conjugated Conjugated Primary Antibodies-bs-0521R-PE; Bioss, USA); (2) double staining with CD34 PE and CD45 PerCP (PerCP-Cy5.5 anti-human cross anti-rabbit CD45, BD Biosciences, USA); (3) double staining with CD34 PE; (4) double staining with CD45 PerCP; and (5) double staining with CD105 fluorescein isothiocyanate (FITC) (Rabbit Anti-Thy-1/CD105/Thy1.1 Polyclonal Antibody, 0778R-FITC, Bioss, USA) in true count tubes. All samples were then stored at 4°C in a dark room and analyzed by flow cytometry for 1 h [21].

### Histopathology observation

The improvement in testicular function with low libido for producing sperm was observed by histopathological preparation by HE staining and calculating the number of spermatogenic cells (spermatogonia, primary spermatocytes, secondary spermatocytes, and spermatid) and Leydig cells. Histopathological examination was performed using a light microscope (Oxford BCM 400, monotaro.id, Indonesia) under magnification of 200×. Observations were made after one cycle of spermatogenesis process (35 days) during which food was provided normally; male rats were operated to collect the testicle tissue. The calculation of the number of spermatogenic cells was based on the total number of four cell types (spermatogonia, primary spermatocytes, secondary spermatocytes, and spermatid) located on the five lumens of the seminiferous tubules from five preparate slides. Furthermore, the total number of the four cell types of the five tubules was divided by 5 [1,2].

The testicular tissue of the rat was fixed in 10% formalin, and 1 h later, mid-testis was injected with 10% formalin. Rats testes then were dehydrated in gradually higher concentration of alcohol, i.e. from 70%, 80%, 90%, and 96%. Then, the testes were cleaned with xylol. Next, the testes were embedded in liquid paraffin and put into molds. Before staining and sectioning, an incision was made using a microtome and mounted on glass slides. Furthermore, staining was performed by removing paraffin with xylol, followed by treating with decreased concentration of alcohol, i.e. from 96%, 90%, 80%, and 70%, and then subjected to HE staining. Finally, mounting was performed after treating the slides with increasing concentration of alcohol, from 70%, 80%, 90%, and 96%, to remove excess stain and then treated with xylol. The preparations were then covered with a cover glass and mounted with Canada balsam (Cas No: 8007-47-4, Sigma Aldrich^®^, Burlington, Massachusetts, USA)[22].

### Statistical analysis

VEGF expression, calculated numbers of CD3^4+^ and CD4^5+^ cells, and number of spermatogenic and Leydig cells were statistically analyzed using SPSS 17 for Windows XP (IBM SPSS Statistic Application, America) with the confidence level of 99% (α=0.01) and level of significance of 0.05 (p=0.05). The steps of hypothesis testing were as follows: normality data were tested by the Kolmogorov–Smirnov test, homogeneity of variance by analysis of variance, and *post hoc* test using the Tukey’s honest significant difference test with 5% as the least significant difference.

## Results

**Figure-1:**
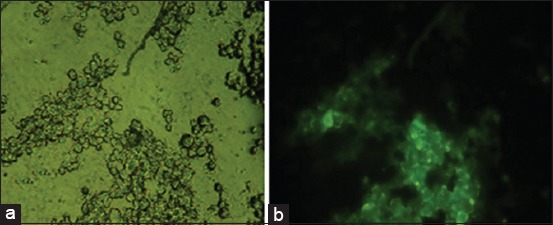
Positive expression of cluster of differentiation (CD)105 in mesenchymal stem cells (MSCs) using a fluorescent microscope at a magnification of 400×. (a) Without filter, MSCs; (b) with green filter, fluorescent MSCs (CD105).

**Figure-2:**
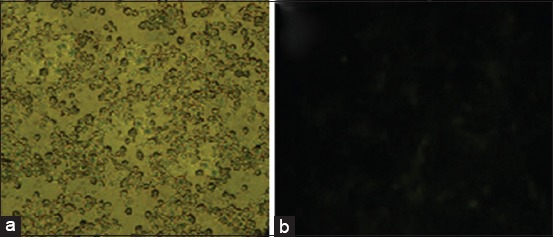
Negative expression of cluster of differentiation (CD)45 in mesenchymal stem cells (MSCs) using a fluorescent microscope at a magnification of 400×. (a) Without filter, MSCs; (b) with green filter, non-fluorescent MSCs (CD45).

The purity of MSCs derived bone marrow was determined by immunofluorescence through the identification of CD105^+^ and CD45^−^ cells [17]. Immunofluorescence identification for CD105 was positive expression and CD45 was negative expression. CD105^+^ and CD45^−^ cells were determined using specific cell surface markers of MSCs using the monoclonal antibody FITC anti-rabbit CD105 (Biolegend) and FITC anti-rabbit CD45 (Biolegend). Immunofluorescence results showed that cultured cells were true MSCs (Figures-1 and 2).

### IHC analysis

**Figure-3:**
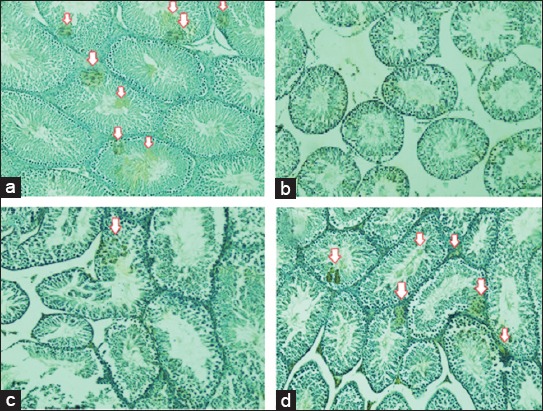
Immunohistochemistry analysis of homing signal of stem cells based on vascular endothelial growth factor (VEGF) expression (brown chromogen) with different treatments at 200× magnification (Nikon H600L Microscope; digital camera DS Fi2 300 megapixel, Nicon Instruments Inc., America). Different superscripts indicate significant difference at p<0.05. (a) Negative control group (T−), rats with normal testicle injected with 0.1 mL of phosphate-buffered saline (PBS) showed VEGF expression of 2.95c±0.40; (b) sham group (TS), rats with testicular failure and low libido injected with 0.1 mL of PBS showed VEGF expression of 0a±0; (c) first group (T1), rats with testicular failure and low libido were injected with 200 million normoxia-conditioned rat mesenchymal stem cells (rMSC-CN)/rat showed VEGF expression of 0.33a±0.48; (d) second group (T2), rats with testicular failure and low libido injected with 200 million hypoxia-conditioned rMSC/rat showed VEGF expression of 2.00b±0.50.

The IHC analysis was performed by scoring 0 to 5 as follows: score 0 means no chromogen colored, scores 1, 2, 3, 4 mean 1-25%, 26-25%, 51-75%, 76-100% chromogenic color expression of VEGF respectively. The average VEGF expression in T2 was 2.00^b^±0.5% (26%-50%) (Figure-3d), although the percentage was lower than that in T−, which was 2.95^c^±0.4% (>50%; Figure-3a), whereas the percentage was still higher than that in T1, with the score being 0.33^a^±0.48% (<5%; Figure-3c). No expression was noted in TS 0^a^±0% (0%; Figure-3b). All scores of VEGF expression are summarized in Table-1.

**Table-1:** Score of VEGF expression by IHC analysis and the calculated number of CD34^+^and CD45^+^cell by flow cytometry in rat testicle tissue after different treatments (mean%±SD).

Treatments	Average VEGF expression score±SD	Average number of calculated CD34^+^and CD45^+^cells (%)±SD
Negative control group (T−): Rats with normal testicle were injected with 0.1 mL of PBS	2.95^c^±0.40	18.25^a^±0.50
Sham group (TS): Rats with testicular failure and low libido were injected with 0.1 mL of PBS	0^a^±0	19.45^a^±0.35
First group (T1): Rats with testicular failure and low libido were injected with 200 million rMSC-CN/rat	0.33^a^±0.48	32.15^b^±1.65
Second group (T2): Rats with testicular failure and low libido were injected with 200 million rMSC-CH/rat	2.00^b^±0.50	83.65^c^±1.50

a,b,c Values in the same column with different superscripts indicate significant difference at p<0.05 (n=10). SD=Standard deviation, VEGF=Vascular endothelial growth factor, IHC=Immunohistochemistry, CD=Cluster of differentiation, rMSC-CH=Hypoxia-conditioned rat mesenchymal stem cells, rMSC-CN=Normoxia-conditioned rat mesenchymal stem cells

### Flow cytometry observation

**Figure-4:**
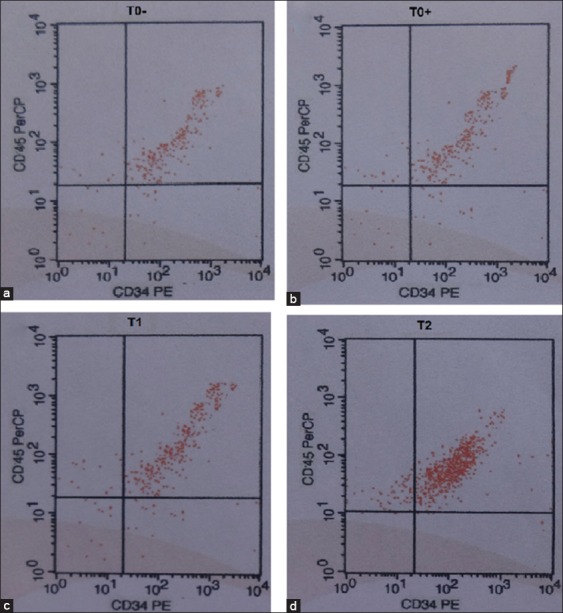
Flow cytometry analysis for hematopoietic stem cell mobilization based on expression of cluster of differentiation (CD)34^+^ and CD45^+^ cells with different treatments. Different superscripts indicate significant difference at p<0.05. (a) Negative control group (T−), rats with normal testis injected with 0.1 mL of phosphate-buffered saline (PBS) showed CD34^+^ and CD45^+^ expression of 18.25a±0.50; (b) sham group (TS), rats with oligospermia and low libido injected with 0.1 mL of PBS showed CD34^+^ and CD45^+^ expression of 19.45a±0.35; (c) first group (T1), rats with oligospermia and low libido injected with 200 million stem cells/rat from high oxygen culture (21% O2) showed CD34^+^ and CD45^+^ expression of 32.15b±1.65; (d) second group (T2), rats with oligospermia and low libido injected with 200 million stem cells/rat from low oxygen culture (1% O2) showed CD34^+^ and CD45^+^ expression of 83.65c±1.50.

Flow cytometry observation of stem cell mobilization was based on the calculated number of CD34^+^, CD45^+^, and CD105^−^ cells. The average number of CD34^+^ and CD45^+^ cells calculated in T− and TS was <20% (Figure-4a and 4b). However, the percentages in T1 were >30% (Figure-4c) and >80% in T2 (Figure-4d), indicating the mobilization of hematopoietic stem cells (HSCs). There was a significant difference (p<0.05) between T2 and the other three groups (T−, TS, and T1) and no difference (p>0.05) between the T− and TS, although both groups were statistically different from T1 (Figure-4 and Table-1).

### Histopathology and libido observation

The improvement in testicular function with oligospermia was observed by histopathological observation based on the number of spermatogenic cells (spermatogonia, primary spermatocytes, secondary spermatocytes, and spermatid). The libido of male rats was assessed based on the number of Leydig cells (Table-2), the function of which is production of testosterone to stimulate libido [21,22]. The results showed that although spermatogenic cell production in T2 decreased significantly (p>0.05) compared to that in T−, and it was significantly (p<0.05) different compared to that in TS and T1 (Table-2). Moreover, it was observed that spermatogenic cell production in T1 was significantly (p<0.05) different compared to that in T− and T2.

**Table-2:** Number of spermatogenic (spermatogonia, primary spermatocytes, secondary spermatocytes, and spermatid) and Leydig cells on several treatments.

Treatments	Average spermatogenic cells (spermatogonia, primary spermatocytes, secondary spermatocytes, spermatid) ± SD	Average Leydig cells±SD
Negative control group (T−): Rats with normal testicle were injected with 0.1 mL of PBS	252.20^d^±2.55	12.50^d^±0.50
Sham group (TS): Rats with testicular failure and low libido were injected with 0.1 mL of PBS	63.35^a^±2.40	2.10^a^±0.40
First group (T1): Rats with testicular failure and low libido were injected with 200 million rMSC-CN/rat	97.35^b^±1.35	5.75^b^±0.30
Second group (T2): Rats with testicular failure and low libido were injected with 200 million rMSC-CH/rat	199.75^c^±1.53	9.25^c^±0.50

a,b,c,d Different superscripts in the same column indicate significant difference (p<0.05). SD, standard deviation; VEGF, vascular endothelial growth factor; PBS, phosphate-buffered saline; rMSC-CH, hypoxia-conditioned rat mesenchymal stem cells; rMSC-CN, normoxia-conditioned rat mesenchymal stem cells

## Discussion

MSCs can differentiate into various cell types, such as osteoblasts, chondrocytes, myocytes, and adipocytes. Currently, however, MSCs can be used to differentiate into germ cell lineage or MSCs that assist in the regeneration of germ cell lineage. There is a phenomenon of transdifferentiation for MSCs, indicating its multipotent characteristics. MSCs can differentiate into various cells, from mesoderm into ectoderm, neuronal cells [23], endoderm [24], and germline cells (spermatogonial, Sertoli, and Leydig cells) and tissues (seminiferous tubules) [25].

The efficacy of rMSC-CH biotechnological culture for testicular failure therapy with low libido was determined based on the following: (a) expression of VEGF as the homing signal, (b) increase in the calculated number of CD34^+^ and CD45^+^ cells, as an indicator of stem cell mobilization, (c) improvement in testis for producing spermatogenic cells, and (d) improvement in libido, based on the number of Leydig cells.

VEGF expression, as a marker of homing signal, was determined through IHC analysis. VEGF is a component of the extracellular matrix of stem cells. Moreover, it plays a role in supporting a conducive microenvironment for stem cells to remain viable [26]. The low oxygen culture used for stem cells in this study provides a supportive niche and triggers expression of VEGF-1, which is a homing signal. Furthermore, VEGF-1 binds to VEGF receptor-1, thus, activating a series of signaling events that activate stem cell factor (SCF). SCF is physiologically vital and is in the niche of protein expression that helps in further communication [27]. The SCF receptor complex progresses into the cell nucleus to activate the expression of nuclear β1-integrin for activating octamer 4 (OCT 4). OCT 4 is a member of the POU family of transcription factors. OCT 4 plays a major role in the proliferation, self-renewal, and differentiation of stem cells. Proliferation changes the state of HSCs from quiescent to the cycling state so that HSCs are shifted from the central endosteum area toward the bone marrow. This suggests that cycling HSCs are found outside their niche and are mobilized to peripheral circulation [28].

Stem cell mobilization could occur because induction of stem cells mobilization is toward the deficient site, based on the calculated number of CD34^+^ and CD45^+^ cells in this study. Mobilization can occur in several ways: (a) proteolytic induction of pharmacological agents, such as granulocyte-colony stimulating factor or cyclophosphamide from bone marrow inside the microenvironment; (b) blockade from specific locking molecules, such as AMD3100, BIO4860CXCR4, or VLA-4; (c) effect of dopamine and β2-adrenergic receptors as neural mediators; (d) element modulation from cascade coagulation; (e) induction of inflammatory signals, such as cytokines, nuclear factor-kappaB (NF-κB), and β catenin, through Wnt due to tissue damage; and (f) homing signals, such as stromal cell-derived factor-1, C-X-C motif chemokine ligand 12 (CXCL12), VEGF, hepatocyte growth factor, platelet-derived growth factor, and integrin, that act for recruitment of stem cells [17]. In this study, as mentioned in point “f”, mobilization occurred due to the expression of homing signal, such as VEGF.

Libido in male rats was assessed by determining the number of Leydig cells. Decreased libido in TS occurred because the adrenal cortex was ineffective in producing DHEA due to malnutrition (rats experienced fasting for five days). Low levels of DHEA in the blood can be a cause of fatigue and decreased body stamina. DHEA is the most potent precursor of steroid hormones, such as testosterone, produced by the renal adrenal cortex [18] and Leydig cells or interstitial cells between seminiferous tubules of testis [3]. In T2, rats with oligospermia and low libido were injected with 200 million stem cells/rat from low oxygen culture (1% O_2_). The results suggested that 200 million stem cells/rat from low oxygen culture (1% O_2_) maintained libido same as that observed in T−. Increased DHEA as a precursor of steroid hormones, such as the hormone testosterone, is also responsible for fat metabolism and acts as an enzyme inhibitor of glucose 6-phosphate dehydrogenase, which acts as a biocatalyst for converting glucose to fat. Thus, increase in DHEA allows for an increase in the amount of free adenosine triphosphate (ATP) in the body and reproduction organ, thus, increasing the stamina of the body [29] and health of reproduction organs [3]. Further, increases in free ATP in the body and reproduction organ in addition to increased stamina also support an increase in libido. It can be explained that libido can be considered suitable if the body is in a state of excellent stamina, thus, allowing the process of reproduction, such as spermatogenesis.

In T1, rats with testicular failure and low libido were injected with 200 million stem cells/rat from high oxygen culture (21% O_2_). The results suggested that 200 million stem cells/rat from high oxygen culture (21% concentration of O_2_) were unable to maintain libido same to that observed in T−. A decrease in Leydig cells reduces testosterone production and leads to decreased libido in males.

The libido process begins with the stimulation of the central nervous system in the hypothalamus, where dopamine is produced as a neurotransmitter and neurohormone that affects sexual behavior and activity in individuals. The stimuli received by the sensory nerve trigger acetaminophen that stimulates endothelial cells to secrete nitric oxide for activating cyclic guanosine monophosphate (cGMP). This process causes the muscles of corpus cavernosum in the penis to swell, leading to the constriction of penis arterioles so that the blood flow is increased. This, in turn, causes the erection of penis due to full blood flow by depressing the veins and inhibiting the release of blood flow so that there is an increase in turgor from the reproduction organs and erection occurs [30].

The lack of glucose in both TS and T1 led to no or less fuel and energy sources, in this case glucose, which is universal for all cells, including spermatogenic and sperm cells. No carbon source is available due to unavailability of glucose for the synthesis of several compounds, such as fatty acids; cholesterol; amino acids; nucleic acids; and steroid hormones, such as testosterone. Glucose is also needed as a precursor to a variety of other sugars, such as lactose, nucleotides, and glycosaminoglycans [31].

Glucose in the cytoplasm of spermatozoa undergoes glycolysis, wherein glucose is catabolized to form pyruvate to produce two molecules of ATP by phosphorylation at the substrate level. In this process, nicotinamide adenine dinucleotide (NAD^+^) is converted to nicotinamide adenine dinucleotide hydrogen (NADH), and NADH is then transferred to the mitochondrial electron chain to form pyruvic acid in the citric acid cycle for complete oxidation to CO_2_. Citric acid cycle, known as the tricarboxylic cycle, is a catabolism reaction path occurring in the matrix of mitochondria. As the process of the citric acid cycle and glycolysis proceeds in the mitochondria inner membrane, oxidative phosphorylation occurs in the mitochondria to use high-energy ATP [32]. The entire process series is known as aerobic glucose oxidation, which produces 38 molecules of ATP [31].

In this study, results of T1 suggested that rats injected with 200 million rMSC-CN/rat from normoxia culture (21% O_2_) were unable to stimulate libido. However, rats injected with 200 million rMSC-CH/rats from hypoxia culture (1% O_2_) increased the number of Leydig cells significantly, which was different from that in TS and T1. However, the increase in Leydig cells in the T2 group was different from that in T− (Table-2).

## Conclusion

The efficacy of rMSC-CH biotechnological culture for testicular failure therapy with low libido was determined based on the expression of VEGF, increase in the number of CD34^+^ and CD45^+^ cells, improvement in the testis to produce spermatogenic cells, and improvement in libido based on the number of Leydig cells.

## Authors’ Contributions

ES: Research project leader, research and ethical clearance preparation, IHC and flow cytometry analysis, stem cell isolation from rat bone marrow, rMSC-CH and rMSC-CN procedure, stem cell transplantation, statistical procedure, and drafting the manuscript preparation (wrote the paper). MH: Rat testicular failure and low libido model, observation of improvement in testicular function based on the calculated number of spermatogenic and sperm cells, observation of libido (based on the number of Leydig cells), designing the study, analyzing statistical data, proofreading, and corresponding author. All the authors have read and approved the final version of the manuscript.
